# New genome assemblies reveal patterns of domestication and adaptation across *Brettanomyces* (*Dekkera*) species

**DOI:** 10.1186/s12864-020-6595-z

**Published:** 2020-03-02

**Authors:** Michael J. Roach, Anthony R. Borneman

**Affiliations:** The Australian Wine Research Institute, PO Box 197, Glen Osmond, South Australia 5046 Australia

**Keywords:** *Brettanomyces*, Genome comparison, Diploid assembly, Wine, Yeast

## Abstract

**Background:**

Yeasts of the genus *Brettanomyces* are of significant interest, both for their capacity to spoil, as well as their potential to positively contribute to different industrial fermentations. However, considerable variance exists in the depth of research and knowledgebase of the five currently known species of *Brettanomyces*. For instance, *Brettanomyces bruxellensis* has been heavily studied and many resources are available for this species, whereas *Brettanomyces nanus* is rarely studied and lacks a publicly available genome assembly altogether. The purpose of this study is to fill this knowledge gap and explore the genomic adaptations that have shaped the evolution of this genus.

**Results:**

Strains for each of the five widely accepted species of *Brettanomyces* (*Brettanomyces anomalus*, *B. bruxellensis*, *Brettanomyces custersianus*, *Brettanomyces naardenensis*, and *B. nanus*) were sequenced using a combination of long- and short-read sequencing technologies. Highly contiguous assemblies were produced for each species. Structural differences between the species’ genomes were observed with gene expansions in fermentation-relevant genes (particularly in *B. bruxellensis* and *B. nanus*) identified. Numerous horizontal gene transfer (HGT) events in all *Brettanomyces* species’, including an HGT event that is probably responsible for allowing *B. bruxellensis* and *B. anomalus* to utilize sucrose were also observed.

**Conclusions:**

Genomic adaptations and some evidence of domestication that have taken place in *Brettanomyces* are outlined. These new genome assemblies form a valuable resource for future research in *Brettanomyces*.

## Background

Most commercial alcoholic fermentations are currently performed by yeast from the genus *Saccharomyces* with the most common species being *Saccharomyces cerevisiae*. The domestication of *S. cerevisiae* is thought to have begun as early as prehistoric times [1]. To date, many commercially available strains have been selected for fermentation in harsh conditions, such as those encountered during wine, beer, and industrial bioethanol fermentations [2–4]. In parallel with *Saccharomyces*, a distantly related genus of budding yeasts, *Brettanomyces* (teleomorph *Dekkera*), has also convergently evolved to occupy this same fermentative niche [5].

There are currently five accepted species of *Brettanomyces*: *B. anomalus*, *B. bruxellensis*, *B. custersianus*, *B. naardenensis*, and *B. nanus* [6]. A sixth species, *Brettanomyces acidodurans*, was recently described, although this species has only been tentatively assigned to this genus, due to a high genetic divergence relative to five species and has not been included in this study [7]. *Brettanomyces* species were originally characterized with a combination of morphological, physiological, and chemotaxonomical traits [8], although the phylogeny has since been defined and updated using several methodologies, often with conflicting results [8–10]. Three different phylogenies were originally presented based on analyses of the 18S or 26S ribosomal RNA sequences, which showed conflicting placement of *B. custersianus* and *B. naardenensis* [8]. Four additional phylogenies, based on either 18S or 26S RNA, or on the concatenated sequences for SSU, LSU, and elongation factor 1α sequences have also been published [9, 10]. These show a consistent placement for *B. custersianus* but somewhat inconsistent branching and poor branch support for *B. naardenensis* and *B. nanus*.

*Brettanomyces spp.* are most commonly associated with spoilage in beer, wine, and soft drink due to the production of many off-flavour metabolites including acetic acid, and vinyl- and ethyl-phenols [5, 11, 12]. However, *Brettanomyces* can also represent an important and favorable component of traditional Belgian Lambic beers [13, 14], and their use has increased in recent years in the craft brewing industry [15]. Furthermore, *B. bruxellensis* has shown potential in bioethanol production by outcompeting *S. cerevisiae* and for its ability to utilize novel substrates [16, 17].

*B. bruxellensis* and to a lesser extent *B. anomalus*, are the main species encountered during wine and beer fermentation and has led to the majority of *Brettanomyces* research focusing only on these two species. The initial assembly of the triploid *B. bruxellensis* strain AWRI1499 [18] has enabled genomics to facilitate research on this organism [19–23]. Subsequent efforts have seen the *B. bruxellensis* genome resolved to chromosome-level scaffolds [24]. In contrast, the assemblies that are available for *B. anomalus* [25], *B. custersianus*, and *B. naardenensis*, are less contiguous, and are mostly un-annotated, while no genome assembly is currently available for *B. nanus*.

*Brettanomyces* genomes have been shown to vary considerable in terms of ploidy and karyotype with haploid, diploid, and triploid strains of *B. bruxellensis* being observed [22, 26]. In addition to ploidy variation, karyotypes can also vary widely, with chromosomal numbers in *B. bruxellensis* being estimated to range between 4 and 9 depending on the strain [27]. Currently available assemblies for *Brettanomyces* vary from 10.2 Mb for *B. custersianus*, and between 11.8 Mb and 15.4 Mb for *B. bruxellensis* (based on haploid genome size).

Recent advancements in third-generation long-read sequencing have enabled the rapid production of highly accurate and contiguous genome assemblies, particularly for microorganisms (reviewed in [28]). This study sought to fill knowledge gaps for various *Brettanomyces* species by sequencing and assembling genomes using current-generation long-read sequencing technologies [29], and then to use these new assemblies to explore the genomic adaptations that have taken place across the *Brettanomyces* genus.

## Results

### New genome assemblies for the *Brettanomyces* genus

Information regarding the species and strains used in this study is listed in Table 1. In the interest of obtaining high-quality and contiguous assemblies, haploid or homozygous strains were favored (the *B. anomalus* strain was the exception), with strains that featured in past studies prioritized. All strains had been isolated from commercial beverage products, with three from commercial fermentations.

**Table 1:** Strain details and growth conditions

ID	Species	Other IDs	Sample origin	Source; Reference
AWRI950	*B. custersianus*	CBS 4805/IFO 1585	Beer	CBS [30];
AWRI951	*B. naardenensis*	CBS 6042/IFO 1588	Soft drink	CBS [31];
AWRI953	*B. anomalus*	CBS 8139	Soft drink	CBS [32];
AWRI2804	*B. bruxellensis*	UCD 2041	Fruit wine	UC Davis Collection
AWRI2847	*B. nanus*	CBS 1945	Beer	CBS [33];

Haploid assemblies were produced for all the *Brettanomyces* species (genome assembly summary statistics are shown in Table 2 and MinION sequencing statistics are available in Table S1). Genome sizes for *B. bruxellensis* and *B. anomalus* of 13.2 and 13.7 Mb, respectively were well within the range of other publicly-available *Brettanomyces* assemblies, which range from 11.8 Mb to 15.4 Mb [18, 24, 25, 34, 35]. The *B. custersianus* assembly size was 10.7 Mb, similar to assemblies of other *B. custersianus* strains (10.2 Mb to 10.4 Mb) [36]. The *B. naardenensis* assembly was 11.16 Mb, highly similar to the only other published assembly [37]. The *B. nanus* assembly was the smallest at only 10.2 Mb and represents in the first whole-genome sequence for this species.

**Table 2:** Assembly and BUSCO summary statistics for the haploid assemblies

	*B. anomalus* (haploid)	*B. anomalus* (diploid)	*B. bruxellensis* (haploid)	*B. custersianus* (haploid)	*B. naardenensis* (haploid)	*B. nanus* (haploid)
Contigs	48	93	12	24	16	5
Length (Mb)	13.77	27.07	13.20	10.73	11.16	10.19
N50 (Mb)	0.640	0.730	2.936	0.847	1.231	3.303
GC (%)	39.81	39.84	39.88	40.24	44.60	41.51
BGs (%)						
Complete	83.0	84.2	88.6	88.2	90.6	90.6
-Single-copy	81.8	48.3	88	87.3	90	90.1
-Duplicate	1.2	35.9	0.6	0.9	0.6	0.5
Fragmented	9.8	8.8	6.1	6.4	5.6	5.2
Missing	7.2	7.0	5.3	5.4	3.8	4.2

The overall contiguity of the assemblies varies due to differences in heterozygosity and sequencing read lengths. The *B. anomalus* strain is a heterozygous diploid organism and while read coverage was high, the median read length was relatively low at 4.7 kb. This resulted in the lowest contiguity in the study consisting of 48 contigs for the haploid assembly with an N_50_ of 640 kb. The *B. nanus* strain is a haploid organism and had a much higher median read length of 14.9 kb. As such, this assembly had the best contiguity consisting of only 5 contigs with an N_50_ of 3.3 Mb.

In order to assess the completeness of each assembly, BUSCO statistics were compiled for each genome (Table 2). Predicted genome completeness was high for the haploid assemblies, with between 3.8% (*B. naardenensis*) and 7.2% (*B. anomalus*) missing BUSCO genes (BGs). The assemblies were then processed with Purge Haplotigs [38] to remove duplicated and artifactual contigs. Duplication was low for not only the homozygous strains but also for the heterozygous *B. anomalus* assembly with between 0.5% (*B. nanus*) and 1.2% (*B. anomalus*) duplicate BGs.

Given the significant differences in the genome sizes within the *Brettanomyces* genus, it was of interest to determine if this size range was due to differences in overall gene number, gene compactness or both. The total number of predicted genes, gene densities (the percent of genome that is genic) and the number of orthogroups with multiple entries were calculated for each *Brettanomyces* genome, in addition to *S. cerevisiae* as a point of comparison (Table S2). *B. nanus* (smallest genome) had the fewest genes (5083), the highest gene density (78.1% genic) and the lowest number of expanded orthogroups (5.2%). Conversely, *B. anomalus* (largest genome) exhibited the highest number of genes (5735), the most ortholog duplicates (10.4%) and the largest proportion of intergenic sequences (62.2% genic).

Given the heterozygous nature of the *B. anomalus* genome, a diploid assembly was also generated for the strain AWRI953. The resultant diploid assembly was approximately twice the size of the haploid assembly and had a slightly improved N_50_ of 730 kb. While the genome size doubled, duplicated BGs only increased from 1.2% for the haploid assembly to 35.9% for the diploid assembly. In an ideal scenario, in which both alleles are faithfully separated, duplicated BGs would be closer to 100%. The low number of duplicated BGs was found to mainly be the result of a number of fragmented gene models being present in one of the two haplomes. It should be noted that while the diploid *B. anomalus* assembly is split into Haplome 1 (H1) and Haplome 2 (H2), these haplomes consist of mosaics of both parental haplotypes. This is an unavoidable artefact of assembly where haplotype switching can randomly occur due to breaks in heterozygosity, and between chromosomes.

### Taxonomy of Brettanomyces

**Fig. 1:**
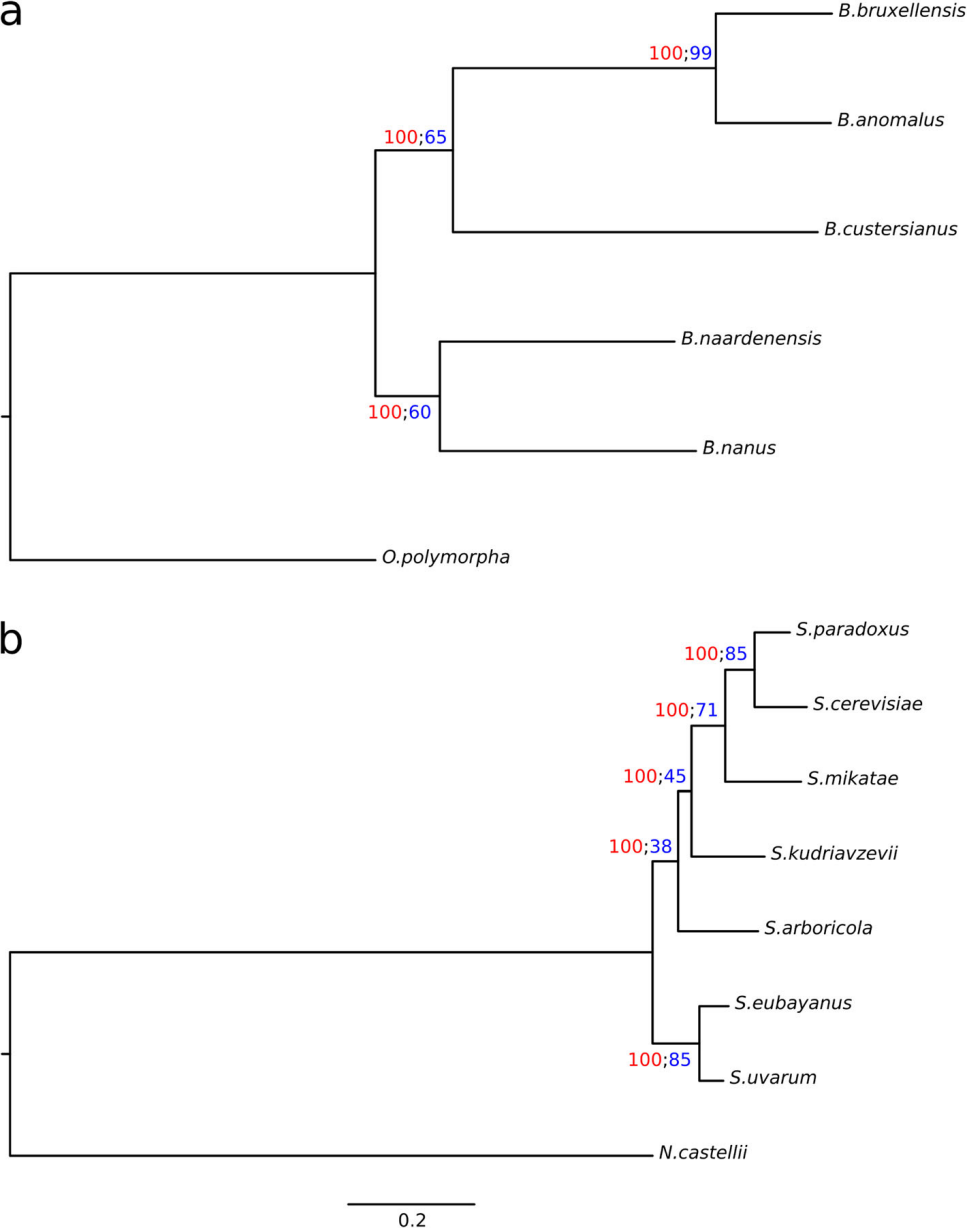
Phylogenies of *Brettanomyces* and *Saccharomyces* species. Rooted, maximum likelihood trees were calculated for *Brettanomyces* species with *Ogataea polymorpha* as an outgroup (**a**) and *Saccharomyces* species with *Naumovozyma castellii* as an outgroup (**b**). The phylogenies were calculated from concatenated codon alignments of single copy orthologs. IQ-TREE’s ultrafast Bootstrap values are calculated from 1000 replications and are shown at branch nodes in red. Branch support calculated from individual gene trees is shown at branch nodes in blue. The two phylogenies are transformed to the same scale (substitutions per site)

This collection of high quality *Brettanomyces* genomes allowed for a comprehensive phylogeny to be generated, which utilized the entire genome, as opposed to extrapolating relationship based upon ribosomal sequences. Codon-based alignments were produced for 3482 single-copy orthologues (SCOs) that were common across the five *Brettanomyces* species, in addition to using *Ogataea polymorpha* (closest available non-*Brettanomyces* genome) as an outgroup. These concatenated alignments were used to calculate a maximum-likelihood tree (Fig. 1a) and to estimate average nucleotide identity (ANI) between pairs of genomes (Table 3). Individual gene trees were also generated for all SCO groups. These individual gene trees were then used to generate a coalescence-based phylogeny (Figure S1a) to check for consistency with, and to generate branch support values for, the concatenation-based phylogeny. As a point of comparison, this phylogenetic methodology was also performed on the members of the *Saccharomyces* genus (Fig. 1b, Figure S1b, and Table 4).

**Table 3:** Average Nucleotide Identities (percent) between *Brettanomyces* species and *Ogataea polymorpha* concatenated single copy ortholog codon alignments

	*B. naardenensis*	*B. bruxellensis*	*B. custersianus*	*O. polymorpha*	*B. anomalus*
*B. nanus*	66.4	60.6	61.0	56.3	60.7
*B. naardenensis*		60.8	61.3	56.6	60.9
*B. bruxellensis*			60.7	55.1	77.1
*B. custersianus*				54.8	60.8
*O. polymorpha*					55.2

**Table 4:** Average Nucleotide Identities (percent) between *Saccharomyces* species and *Naumovozyma castellii* concatenated single copy ortholog codon alignments

	*S. eubayanus*	*S. uvarum*	*S. cerevisiae*	*N. castellii*	*S. paradoxus*	*S. mikatae*	*S. kudriavzevii*
*S. arboricola*	82.1	82.4	81.1	61.6	81.9	81.3	83.2
*S. eubayanus*		92.8	79.9	61.6	80.6	80.1	81.8
*S. uvarum*			80.1	61.5	80.9	80.3	82.2
*S. cerevisiae*				61.6	89.3	84.0	81.9
*N. castellii*					61.6	61.6	61.4
*S. paradoxus*						85.2	82.8
*S. mikatae*							82.2

When compared to the distances between the members of the genus *Saccharomyces*, there is a much larger genetic distance separating the various *Brettanomyces* species. Indeed, there is a greater genetic distance between most of the *Brettanomyces* species than there is between any of the individual *Saccharomyces* species and the outgroup used for that phylogeny (*Naumovozyma castellii*). The largest separation was observed between *B. nanus* and *B. bruxellensis*, which presented an ANI of only 60.6%. The closest relationship between any two *Brettanomyces* species was between *B. bruxellensis* and *B. anomalus* with an ANI of 77.1%, followed by *B. nanus* and *B. naardenensis* with an ANI of 66.4%. The remainder of pairwise ANIs ranged between 60.6 and 61.3%. For comparison, pairwise ANIs calculated between each of the *Saccharomyces* species and the outgroup (*N. castellii*) ranged between 61.4% (*S. kudriavzeviiI*) and 61.6% (*S. cerevisiae*). Furthermore, the genetic distance between the most distantly related *Saccharomyces* species (*S. cerevisiae* and *S. eubayanus*, ANI of 79.9%) is less than the genetic distance between the most closely related *Brettanomyces* species.

### Extensive rearrangements are present throughout *Brettanomyces* genomes

**Fig. 2:**
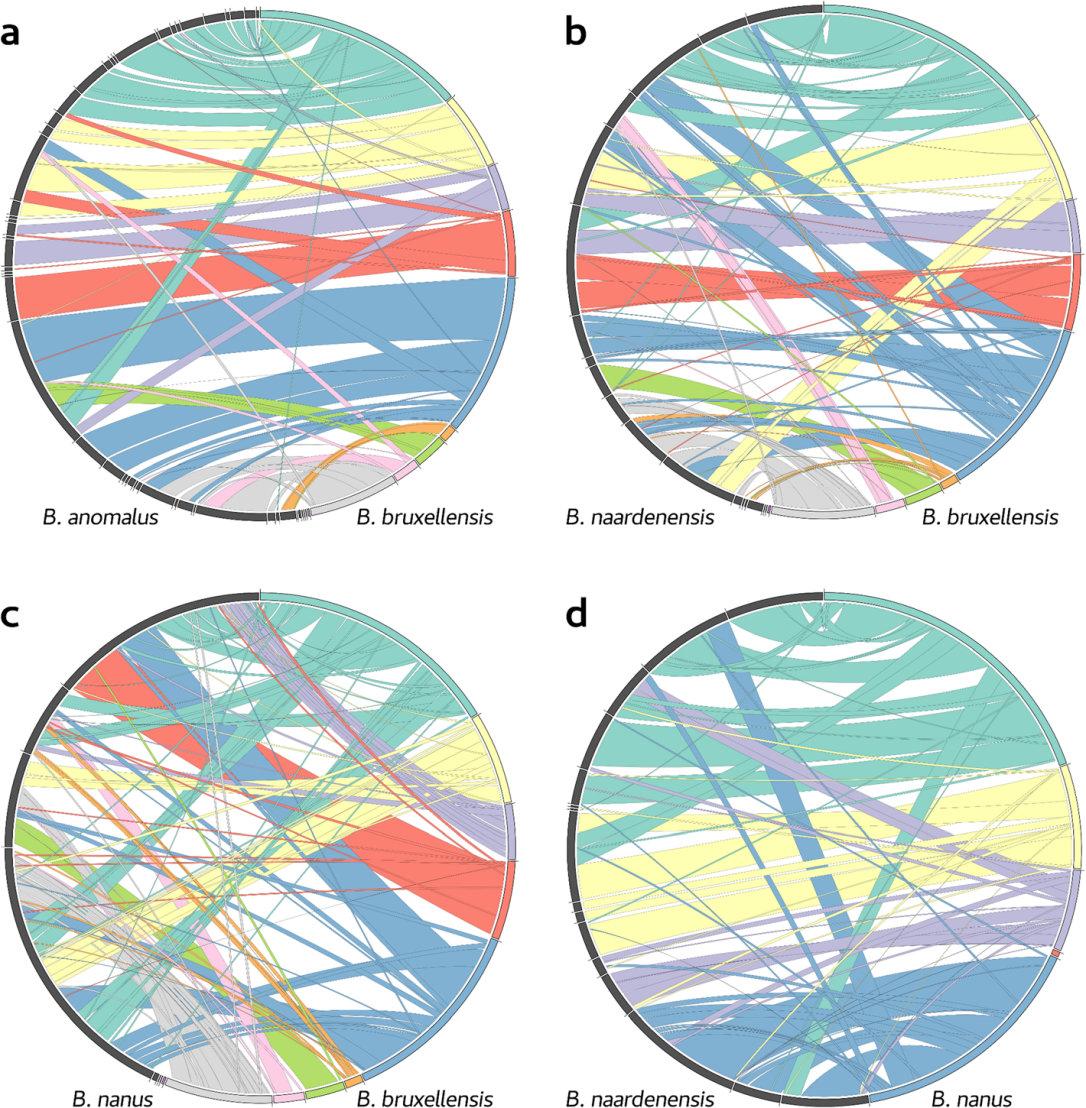
Synteny between haploid assemblies of *Brettanomyces*, visualized as Circos plots. Reference assembly Contigs are coloured sequentially. Alignments are coloured according to the reference assembly contigs and are layered by alignment length. The query assembly contigs are coloured grey. Alignments are depicted between *B. bruxellensis* and *B. anomalus* (**a**), *B. bruxellensis* and *B. naardenensis* (**b**), *B. bruxellensis* and *B. nanus* (**c**), and *B. nanus* and *B. naardenensis* (**d**)

In order to ascertain if larger-scale differences accompanied the extensive nucleotide diversity that was observed between the *Brettanomyces* species, whole-genome alignments were used to detect structural rearrangements between the genomes (Fig. 2). There were numerous small and several large translocations present between the *B. bruxellensis* and the *B. anomalus* assemblies (Fig. 2a) with a total of 71 syntenic blocks identified. The *B. bruxellensis* and *B. custersianus* assemblies showed less overall synteny, with the alignment broken into 93 syntenic blocks (although individual translocation units appear to be smaller; Figure S2). Comparing *B. bruxellensis* to the more distantly related species *B. naardenensis* (Fig. 2b) and *B. nanus* (Fig. 2c), these breaks in synteny are also common, with 91 and 117 syntenic blocks observed, respectively. The chromosomal rearrangements were also not limited to a single species or clade; when comparing *B. nanus* to *B. naardenensis* (Fig. 2d) there were 73 syntenic blocks identified, very similar to that occurring between *B. bruxellensis* and *B. anomalus*.

Given the heterozygous nature of the *B. anomalus* genome analyzed in this study, the genome was examined for the presence of large LOH tracts. Three large contigs, comprising 2.14 Mb (15%) of the *B. anomalus* genome, were predicted to be homozygous (0.0353 SNPs/kb) while the rest of the genome is heterozygous (3.21 SNPs/kb) (Figure S3). The strains used in this study as reference for *B. bruxellensis, B. custersianus*, *B. naardenensis*, and *B. nanus* appeared homozygous as expected, with heterozygous SNP densities ranging from 0.01 SNPs/kb (*B. naardenensis)* to 0.05 SNPs/kb (*B. bruxellensis)*.

### Enrichment of fermentation-relevant genes

**Fig. 3:**
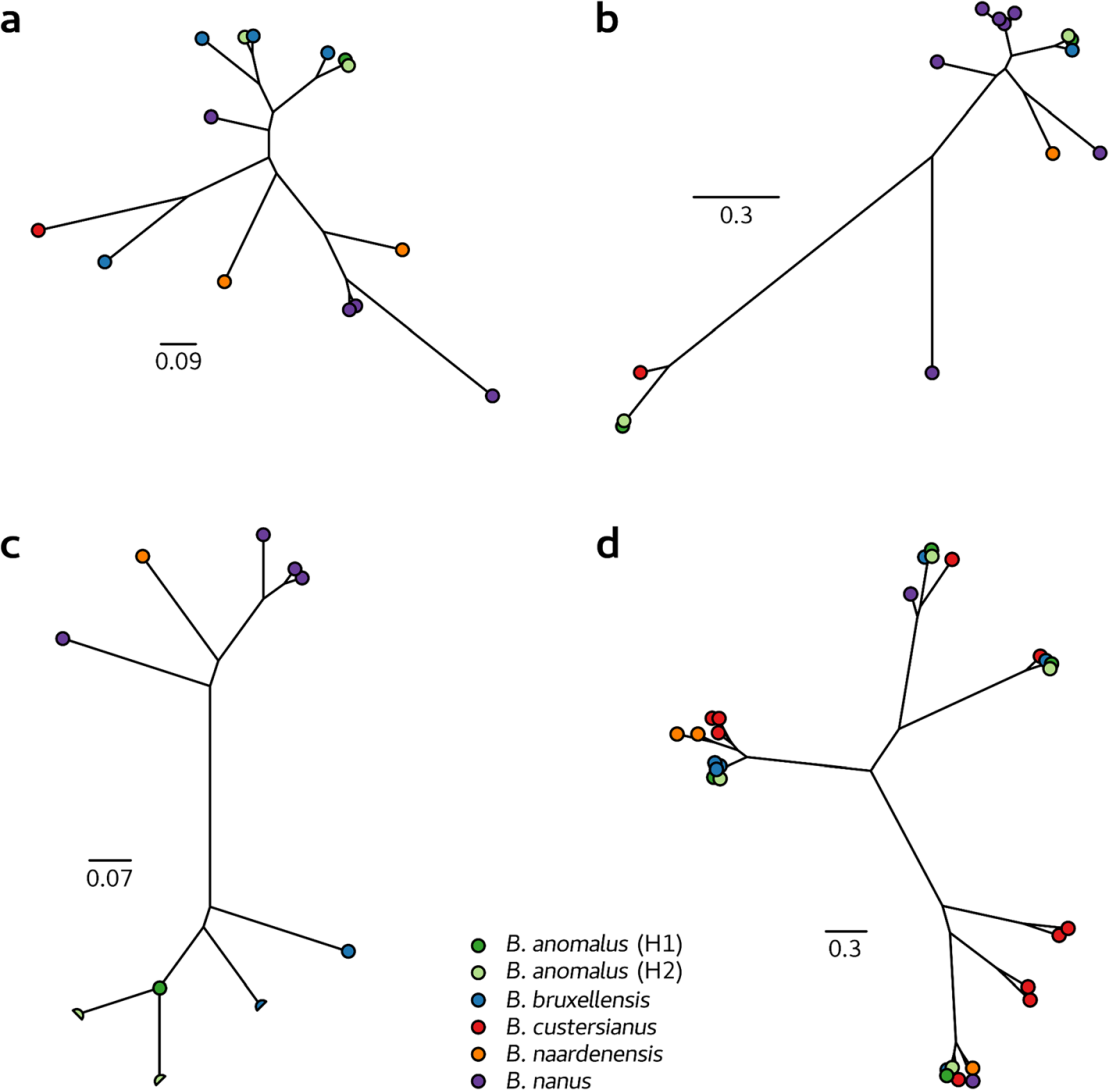
Phylogenies of several enriched orthogroups in *Brettanomyces*. Broken gene models or pseudo-genes are indicated as half circles. The enriched gene orthogroups are: oligo-1,6-glucosidase (EC 3.2.1.10) (**a**), β-glucosidase (EC 3.2.1.21) (**b**), β-galactosidase (EC 3.2.1.23) (**c**), and sarcosine oxidase (EC 1.5.3.1/1.5.3.7) (**d**). Phylogenies are scaled by substitutions per site

Given the apparent adaptation of *Brettanomyces* to the fermentative environment, each *Brettanomyces* genome was investigated for the presence of specific gene family expansions (Table 5). Both *B. bruxellensis* and *B. nanus* were predicted to have undergone copy number expansion of ORFs predicted to encode oligo-1,6-glucosidase enzymes (EC 3.2.1.10), which are commonly associated with starch and galactose metabolism (Fig. 3a). *B. nanus* was also predicted to possess an expanded set of genes encoding β-glucosidase (EC 3.2.1.21; Fig. 3b) and β-galactosidase (EC 3.2.1.23; Fig. 3c) activities, which are involved in the utilization of sugars from complex polysaccharides.

**Table 5:** Expanded gene families in *Brettanomyces*

Species	Gene Name	Count	KEGG ID	KEGG Pathway(s)
*B. anomalus*	formate dehydrogenase	4	K00122	Glyoxylate and dicarboxylate metabolism; Methane metabolism
*B. bruxellensis*	oligo-1,6-glucosidase	4	K01182	Galactose metabolism; Starch and sucrose metabolism
	S-formylglutathione hydrolase	5	K01070	Methane metabolism
*B. custersianus*	NADPH2 dehydrogenase	4	K00354	–
	sarcosine oxidase/L-pipecolate oxidase	5	K00306	Peroxisome; Glycine, serine and threonine metabolism; Lysine degradation
*B. naardenensis*	acetylornithine deacetylase	3	K01438	Arginine biosynthesis
	NADPH2 dehydrogenase	5	K00354	–
	sulfonate dioxygenase	5	K19245	–
*B. nanus*	oligo-1,6-glucosidase	3	K01182	Galactose metabolism; Starch and sucrose metabolism
	β-galactosidase	4	K01190	Galactose metabolism; Other glycan degradation; Sphingolipid metabolism
	NADPH2 dehydrogenase	6	K00354	–
	β-glucosidase	7	K05349	Phenylpropanoid biosynthesis; Starch and sucrose metabolism; Cyanoamino acid metabolism

*B. custersianus* and *B. bruxellensis* presented large expansions (10 and 6 copies respectively) of genes encoding sarcosine oxidase / L-pipecolate oxidase (PIPOX) (EC 1.5.3.1/1.5.3.7) and the remaining *Brettanomyces* species also contained multiple copies of this gene (Fig. 3d). PIPOX exhibits broad substrate specificity, but primarily catalyzes the breakdown of sarcosine to glycine and formaldehyde, in addition to the oxidation of L-pipecolate [39]. It has been shown that PIPOX also acts on numerous other *N*-methyl amino acids such as *N*-methyl-L-alanine, *N*-ethylglycine, and more importantly from a winemaking perspective, both L- and D-proline [39–42].

In addition to PIPOX, *B. bruxellensis* and *B. anomalus* share an expansion of S-formylglutathione hydrolase (EC 3.1.2.12), and *B. anomalus* contains an expansion of formate dehydrogenase (EC 1.17.1.9). These genes are part of methanol metabolism in other species (a capability lost in *Brettanomyces*) and are also involved with the metabolism of formaldehyde (a common metabolite during fermentation). Lastly, *B. naardenensis* contains an expansion of a gene encoding sulfonate dioxygenase (EC 1.14.11.-) activity, associated with the utilization of alternative sulphur sources, and an expansion of acetylornithine deacetylase (EC 3.5.1.16), a component of the arginine biosynthetic pathway.

### Horizontal gene transfer enables sucrose utilization in *B. bruxellensis* and *B. anomalus*

Potential HGT events that may have contributed to the evolution of *Brettanomyces* were investigated. Twelve *Brettanomyces* orthogroups were predicted to be the result of HGT from bacteria (Table 6). Of these bacterially derived gene families, a Glycoside Hydrolase family 32 gene (GH32), which was predicted to have β-fructofuranosidase activity (EC 3.2.1.26), is likely to have had a key phenotypic impact during the evolution of this genus. GH32 enzymes hydrolyse glycosidic bonds and β-fructofuranosidase (Invertase) is specifically responsible for the breakdown of sucrose into fructose and glucose monomers and is required for the utilization of sucrose as a carbon source.

**Table 6:** Genes predicted to occur in *Brettanomyces* via Horizontal Gene Transfer

Orthogroup (Saccharomycetaceae)	Gene name	KEGG	Species (Gene ID)	Alien Index	Closest BLAST hit
OG0000714	glutamine amidotransferase	–	*B. nanus* (g3549)	117	*Cyanobacterium*
			*B. naardenensis* (g138)	99	*Cyanobacterium*
OG0001026	α/β hydrolase	–	*B. naardenensis* (g5185)	82	*Klebsiella*
OG0003977	nitronate monooxygenase	–	*B. naardenensis* (g5218)	51	*Halomonas*
OG0003998	β-fructofuranosidase (invertase)	K01193	*B. anomalus* H1 (g3595)	31	*Asaia*
			*B. anomalus* H2 (g273)	29	*Asaia*
			*B. bruxellensis* (g1543)	35	*Asaia*
OG0005068	NADP oxidoreductase	–	*B. naardenensis* (g4928)	37	*Halomonas*
			*B. naardenensis* (g5192)	50	*Halomonas*
			*B. naardenensis* (g5211)	47	*Halomonas*
OG0005439	flavodoxin family protein	K08071	*B. naardenensis* (g1784)	74	*Gluconobacter*
OG0005699	cysteine hydrolase	–	*B. naardenensis* (g5191)	47	*Pseudomonas*
OG0005912	capsule biosynthesis protein CapA	–	*B. anomalus* H1 (g1924)	66	*Izhakiella*
			*B. bruxellensis* (g4262)	56	*Izhakiella*
			*B. custersianus* (g2790)	63	*Izhakiella*
			*B. nanus* (g654)	119	*Izhakiella*
OG0006081	S-antigen protein	–	*B. naardenensis* (g3365)	51	*Arthrospira*
OG0006556	NAD(P)-dependent oxidoreductase	–	*B. anomalus* H1 (g109)	50	*Mycobacteroides*
			*B. anomalus* H2 (g1567)	128	*Clostridium*
			*B. bruxellensis* (g2456)	140	*Clostridium*

**Fig. 4:**
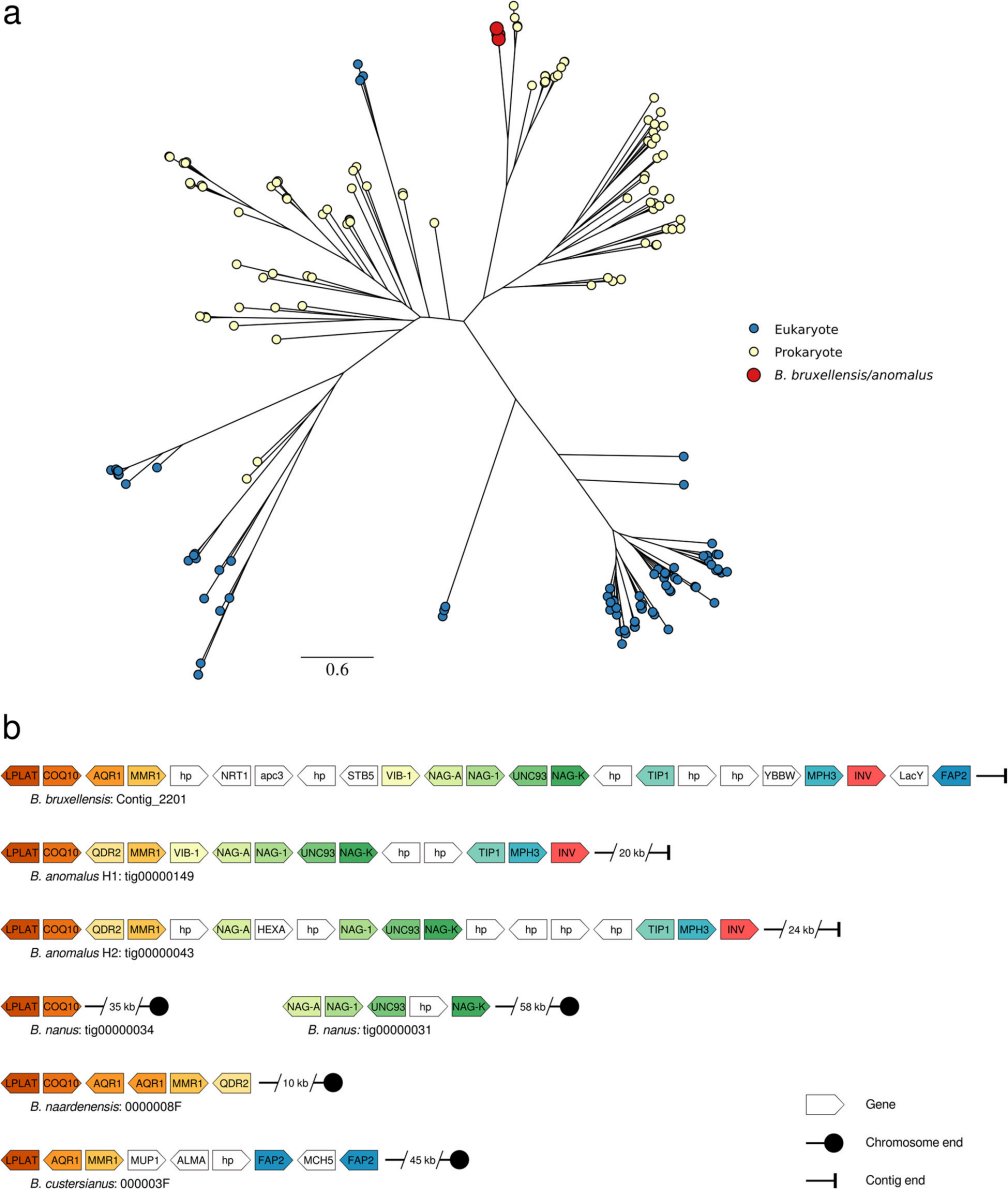
β-fructofuranosidases (invertases) from *Brettanomyces*. Phylogeny of invertases from *Brettanomyces* and the top blast hits from the RefSeq non-redundant prokaryote and eukaryote databases, scaled by substitutions per site, with *Brettanomyces* nodes enlarged for clarity (**a**). Genomic context of invertases in *Brettanomyces*, showing cluster of conserved genes, orange; NAG gene cluster, green; cluster of metabolic genes, blue; Invertase, red (**b**)

To further confirm the bacterial origins of the *Brettanomyces* invertases, a protein-based phylogeny was created from the highest scoring eukaryote and prokaryote blast hits from the RefSeq non-redundant database, as well as from these three *Brettanomyces* invertases (Fig. 4a). The prokaryote and eukaryote invertases each form two distinct clades. Consistent with a bacterial-derived HGT event, the *Brettanomyces* invertase proteins reside within one of the two prokaryote clades and are evolutionarily distinct from the eukaryote groups. There are also three other eukaryote invertases that reside within a prokaryote clade, and two prokaryote invertases that reside within a eukaryote clade, which suggests that HGT of this important enzyme activity is not unique to *Brettanomyces*. To confirm the placement of the *Brettanomyces* invertases in the prokaryotic clade, three alternate topologies (within either of the eukaryote clades, as well as within the second prokaryote clade) were tested (Figure S4, Table S3). These constrained topologies were all significantly less likely compared to the unconstrained tree (Figure S4, Table S3).

The genomic context of the invertases present in *B. bruxellensis* and *B. anomalus* was also examined. These genes are predicted to reside within sub-telomeric regions (Fig. 4b). In *Brettanomyces*, there is significant structural variation and a general loss of synteny, which is typical of sub-telomeric regions in other species (Fig. 4b). For example, in *B. nanus* the NAG gene cluster resides within a different sub-telomere relative to *B. bruxellensis* and *B. anomalus*. The NAG genes are also present in *B. naardenensis,* but are not co-located and appear to be missing entirely in *B. custersianus*. Likewise, homologues of the *MPH3* and *TIP1* genes that are present across all the *Brettanomyces* species, are only found in this specific sub-telomeric region in *B. bruxellensis* and *B. anomalus*.

## Discussion

New genome assemblies for the five *Brettanomyces* species are described, which generally exhibit significant improvements over previous assemblies produced for this genus. The most contiguous genome assembly described was that of *B. nanus*, which comprised only 5 contigs and which had an N_50_ of 3.3 Mb. To the best of our knowledge, this makes the *B. nanus* assembly the most contiguous *Brettanomyces* assembly to date. When comparing the assemblies of the other species to the next most contiguous assembly available from other *Brettanomyces* sequencing studies, the *B. anomalus* assembly represents a 4.7-fold improvement over GCA_001754015.1 (261 contigs), *B. custersianus* a 9.4-fold improvement over GCA_001746385.1 (226 contigs) and *B. naardenensis* and 6.5-fold improvement over GCA_900660285.1 (104 contigs). While the predicted completeness for these new assemblies were all generally high, there was also considerable differences in gene density and content. This was most prominent between *B. nanus* and *B. anomalus*, with the *B. nanus* genome containing fewer total genes, less intergenic sequence and lower duplication of specific orthogroups.

The high-quality genome sequences allowed for the calculation of a *Brettanomyces* whole-genome phylogeny. The topology of the whole-genome phylogeny generally agreed with those derived from rRNA sequences in the placement of *B. bruxellensis*, *B. anomalus* and *B. custersianus* [8–10]. However, these earlier studies were not able to consistently resolve the placement of *B. nanus* and *B. naardenensis*, with conflicting results between phylogenies based on 18S and 26S ribosomal RNA sequences. The whole-genome phylogeny proposes the *Brettanomyces* genus to be comprised of two clades, with *B. nanus* and *B. naardenensis* forming a clade separate from the other species. This whole-genome topology is consistent with previous 18S phylogenies. Comparison of ANI values identified that there is a larger genetic distance separating some *Brettanomyces* species than there was separating the *Saccharomyces* and *Naumovozyma* genera. While ANI values alone are generally insufficient for determining genus boundaries (at least in prokaryotes) [43], the extremely low ANIs that have been observed across the *Brettanomyces* genus merits further consideration into the taxonomy of this group and whether it may be appropriate for the *Brettanomyces* genus to be refined.

*B. nanus*, and to a lesser extent *B. bruxellensis* exhibited expansions of families of glucosidases and galactosidases that are responsible for the utilization of sugars from complex polysaccharides. These types of expansions are a hallmark of the domestication of beer and wine strains of *S. cerevisiae* and suggests that a similar process may be occurring in *B. nanus* [44–46]. The three known *B. nanus* strains were all isolated from beer samples obtained from Swedish breweries in 1952. The *B. nanus* strain AWRI2847 (CBS 1945) was found to have far less spoilage potential in beer than either *B. bruxellensis* or *B. anomalus* [47]. At the time this strain was isolated, microbial spoilage of beer was determined sensorially and sharing yeast samples between both individual fermentations (re-pitching) and breweries was common practice [48]. Taken together, these practices may have allowed *B. nanus* to remain a long-term undetected contaminant, surviving successive serial re-pitchings and spreading to multiple breweries.

The ability of *Brettanomyces* to grow in nutrient-depleted conditions has largely been attributed to the utilization of alternative nitrogen sources such as free nitrates and amino acids [49–51]. The expansion of PIPOX in *Brettanomyces* may be partly responsible for this important survival trait. Proline, a substrate of PIPOX, is one of the more common amino acids in fermented wine and beer. Despite this abundance, proline is poorly utilized by *S. cerevisiae*, however it is readily metabolized by *B. bruxellensis* [52–55]. PIPOX converts proline to 1-pyrroline-2-carboxylate, which can be further converted to D-Ornithine by a general aminotransferase. Unlike proline oxidase (EC 1.5.1.2) and proline dehydrogenase (EC 1.5.5.2) which convert proline to 1-pyrroline-5-carboxylase, PIPOX represents an alternative avenue for proline utilization as a nitrogen source that is less impactful to redox homeostasis, which may allow its utilization during fermentation.

Horizontal Gene Transfer (HGT) has been reported as a mechanism of adaptative evolution in fungal species and to have contributed to the domestication of *S. cerevisiae* [56–58]. Similarly, an HGT event is predicted to have conferred the ability to utilize sucrose as a carbon source to *B. bruxellensis* and *B. anomalus* via the incorporation of a bacterially-derived invertase. Previous phenotypic testing has shown *B. bruxellensis* and *B. anomalus* to be the only *Brettanomyces* species capable of utilizing sucrose [6] and this phenotype correlates with the presence of this HGT-derived invertase, which is only observed in the *B. bruxellensis* and *B. anomalus* genomes (there are no other invertase encoding ORFs predicted in *Brettanomyces*). The genomic context illustrates further parallels to evolution in *Saccharomyces*. The invertases are shown to reside within sub-telomeres, which are genomic regions that have been shown to be hotspots for structural rearrangements and HGT events in *Saccharomyces* [59–63]. Sucrose utilization likely conferred a significant advantage in fruit fermentations, helping to shape the evolution of the common ancestor of *B. bruxellensis* and *B. anomalus* towards this fermentation specialization.

## Conclusions

High quality genome assemblies for all five currently accepted *Brettanomyces* species are described, including the first assembly for *B. nanus* and the most contiguous assemblies available to date for *B. anomalus*, *B. custersianus*, and *B. naardenensis*. Comparative genome analysis established that the species are genetically distant and polyphyletic. Numerous indicators of domestication and adaptation in *Brettanomyces* were identified with some notable parallels to the evolution of *Saccharomyces*. Structural differences between the genomes of the *Brettanomyces* species and apparent loss of heterozygosity in *B. anomalus* were observed. Enrichments of fermentation-relevant genes were identified in *B. anomalus*, *B. bruxellensis* and *B. nanus*, as well as multiple horizontal gene transfer events in all *Brettanomyces* genomes, including a gene in the *B. anomalus* and *B. bruxellensis* genomes that is probably responsible for these species’ ability to utilize sucrose.

## Methods

Detailed workflows, custom scripts for computational analyses and genome annotations are available at https://github.com/mroach-awri/BrettanomycesGenComp (DOI: 10.5281/zenodo.3632185). All sequencing reads and genome assemblies have been deposited at the National Center for Biotechnology Information (NCBI) Sequence Read Archive (SRA) under the BioProject: PRJNA554210. Raw FAST5-format files for all Oxford Nanopore sequencing are available from the European Bioinformatics Institute (EMBL-EBI) European Nucleotide Archive (ENA) under the study: ERP116386.

### Strains and media

The five *Brettanomyces* strains selected for sequencing were supplied by the Australian Wine Research Institute’s wine microorganism culture collection. AWRI953 and AWRI2804 were grown in MYPG medium (0.3% malt extract, 0.3% yeast extract, 0.2% peptone, 1% glucose) at 27 °C and AWRI950, AWRI951, and AWRI2847 were grown in GPYA+CaCO_3_ medium (4% glucose, 0.5% peptone, 0.5% yeast extract, 1% calcium carbonate) at 25 °C.

### Library preparation and sequencing

Genomic DNA was extracted from liquid cultures using a QIAGEN Gentra Puregene Yeast/Bact Kit. *B. bruxellensis* was sequenced using PacBio RS-II SMRT sequencing. The sequencing library for *B. nanus* was multiplexed with other samples (not reported here) using the SQK-LSK109 and EXP-NBD103 kits following the Oxford Nanopore protocol NBE_9065_v109_revA 23MAY2018. For the remaining species, libraries were prepared using the SQK-LSK108 kit following the protocol GDE_9002_v108_revT_18OCT2016. Sequencing was performed on a MinION using FLO-MIN106 flow-cells. Demultiplexing and base-calling were performed using Albacore v2.3.1.

Illumina sequencing was performed on each strain using a combination of short-insert (TruSeq PCR-free) and mate-pair (2-5 kb insert and 6–10 kb insert) libraries. All libraries were barcoded and pooled in a single Miseq sequencing run using 2x300bp chemistry.

### Assembly

The *B. bruxellensis* genome in this study was assembled with Mira v4.9.3 [64] (job = genome,denovo,accurate; −NW:cac = warn; PCBIOHQ_SETTINGS; −CO:mrpg = 7) using PacBio long-reads that were error corrected with Illumina paired-end and mate-pair reads using PBcR (wgs-8.3rc1) [65] with default parameters. This assembly was manually finished in DNASTAR SeqMan Pro. Haploid assemblies for all other *Brettanomyces* species were generated from FASTQ-format Nanopore reads using Canu v1.7 [66]. The Nanopore reads were mapped to the assemblies using minimap2 [67] and initial base-call polishing was performed with Nanopolish v0.9.2 [68], utilizing the FAST5 signal-level sequencing data. Further base-call polishing was performed with Illumina paired-end, and 2–4 kb and 6–10 kb mate-pair reads. Paired-end and mate-pair reads were mapped with BWA-MEM v0.7.12-r1039 [69] and Bowtie2 v2.2.9 [70] respectively; base-call polishing was then performed with Pilon v1.22 [71]. Finally, raw Nanopore reads were mapped to the base-call-polished assemblies and Purge Haplotigs v1.0.1 [38] was used to remove any duplicate or artefactual contigs.

A diploid assembly for AWRI953 (*B. anomalus*) was also generated. Paired-end reads were mapped to the haploid assembly with BWA-MEM, and high-confidence SNPs were called using VarScan v2.3.9 [72]. Nanopore reads were mapped to the assembly using BWA-MEM. Heterozygous SNPs were phased using the mapped Nanopore reads with HapCut2 commit: c2e6608 [73] and converted to VCF format with WhatsHap v0.16 [74]. New consensus sequences were called for each haplotype from the phased SNPs and the nanopore reads were binned according to which haplotype they mapped best. The two *B. anomalus* haplotypes were then independently reassembled from the haplotype-binned nanopore reads using the method described for the other species.

All other *Brettanomyces* assemblies were aligned to the *B. bruxellensis* assembly using NUCmer (MUMmer) v4.0.0beta2 [75]. Dotplots were visualized and contigs with split alignments were manually inspected for indications of mis-assemblies using mapped alignments of Nanopore reads and Illumina mate-pair reads. Genome metrics were calculated with Quast [76] and completeness, duplication, and fragmentation were estimated using BUSCO v3.0.2 [77] with the odb9 Saccharomyceta dataset.

### Annotation

Gene models were predicted with Augustus v3.2.3 [78] using the *S. cerevisiae* S288C configuration. Gene models were submitted for KEGG annotation using BlastKOALA [79], and GO-terms and functional domains were annotated using InterProScan v5.32–71.0 [80]. Orthogroups were assigned with OrthoFinder v2.2.6 [81] using representative species from Saccharomycetaceae (Table S4) and also using only the haploid *Brettanomyces* assemblies.

### Phylogeny

Orthofinder (*Brettanomyces* + *O. polymorpha*) was used to find SCOs over these genomes. Protein sequences were aligned with Muscle v3.8.31 [82] and then converted to codon-spaced alignments using PAL2NAL [83]. Average nucleotide identities were estimated using panito commit: f65ba29 (github.com/sanger-pathogens/panito). A rooted maximum likelihood phylogeny was generated with IQ-TREE [84] on the concatenated codon alignments. IQ-TREE was also used to generate gene trees for all SCOs, and then to generate a coalescence-based phylogeny from the SCO individual gene trees. Phylogenies were created using the same method for the *Saccharomyces* species + *N. castellii* (outgroup) to serve as a comparison.

### Whole genome synteny visualization

Pairwise synteny blocks were generated between the reference *B. bruxellensis* assembly and the other haploid assemblies, as well as between the *B. naardenensis* and *B. nanus* assemblies. Contigs were placed in chromosome order using Purge Haplotigs [38] to generate placement files that were then used to rearrange contigs. Alignments between the assemblies were calculated using NUCmer with sensitive parameters (−b 500 -c 40 -d 0.5 -g 200 -l 12). Genome windows (20 kb windows, 10 kb steps) were generated for the assemblies and a custom script was used to pair syntenic genome windows based on the NUCmer alignments. Concordant overlapping and adjacent windows were merged, and overlapping discordant windows were trimmed. The synteny blocks were then visualized using Circos v0.69.6 [85].

### Gene enrichment

OrthoFinder (Saccharomycetaceae) annotations were used to identify gene-count differences between the *Brettanomyces* species. The ratio of the gene-count to the average gene-count was calculated for the *Brettanomyces* species over all OrthoFinder orthogroups. All orthogroups with a ratio ≥ 2 for any *Brettanomyces* species were subject to GO-enrichment analysis using BiNGO v3.0.3 [86] using the hypergeometric test with Bonferroni Family-Wise Error Rate (FWER) correction. Genes for overrepresented categories (*p*-value ≤0.05) were returned. Multiple sequence alignments were generated for GO-enriched orthogroups using Muscle and maximum likelihood phylogeny trees generated using PhyML within SeaView v4.7 [87] using default parameters (LG model, BioNJ starting tree, tree searching using NNI substitutions).

### Horizontal gene transfer

HGT events were predicted for the *Brettanomyces* species. Protein sequences for the assemblies were used in BLAST-P searches against the RefSeqKB non-redundant Fungi and Bacteria datasets [88], the Alien Index was calculated as described in [89]. All *Brettanomyces* proteins with an AI score greater than 20 were investigated further. The multiple sequence alignments and trees were retrieved for the HGT candidates’ orthogroups and several candidates were removed following manual inspection. A phylogeny was generated for one HGT prediction of interest. The *Brettanomyces* genes, and the top blast hits from the ResSeq non-redundant database eukaryote and prokaryote datasets were aligned with Muscle, and the phylogeny was generated with IQ-TREE. Constrained trees were generated to test the *Brettanomyces* genes within alternate clades and these were assessed using IQ-TREE’s tree topology tests.

## Supplementary information

**Additional file 1: Table S1**. MinION sequencing metrics for *Brettanomyces* sequencing, **Table S2**: Predicted genes, gene density, and orthogroup duplicity for the *Brettanomyces* genomes, **Table S3**: Constrained tree topology tests for *Brettanomyces* invertases (depicted in Figure S4), **Table S4**: Saccharomycetaceae species used with *Brettanomyces* species in OrthoFinder

**Additional file 2: Figure S1.** Coalescences-based phylogenies of *Brettanomyces* and *Saccharomyces*. Rooted, unscaled coalescence-based phylogenies were calculated from individual gene trees of all single copy orthologs for the *Brettanomyces* genus + *Ogataea polymorpha* (*a*), and for the *Saccharomyces* genus + *Naumovozyma castellii* (*b*).

**Additional file 3: Figure S2.** Synteny between haploid assemblies of *B. bruxellensis* and *B. custersianus*, visualized as a Circos plot. Reference assembly Contigs are coloured sequentially. Alignments are coloured according to the reference assembly contigs and are layered by alignment length. The query assembly contigs are coloured grey.

**Additional file 4: Figure S3.** Read-depth and SNP density over haploid assembly of *B. anomalus*, visualized as a Circos plot. Contigs arranged by length (*i*), read-coverage histogram (blue, median coverage; red, low/high coverage) (*ii*), SNP-density (red, low; blue, high) (*iii*).

**Additional file 5: Figure S4.** Constrained topology tests for *Brettanomyces* invertase genes. The unconstrained phylogeny (depicted in Fig. 4) of invertases from *Brettanomyces* and the top blast hits from the RefSeq non-redundant prokaryote and eukaryote databases, scaled by substitutions per site, with *Brettanomyces* nodes enlarged for clarity (*a*). *Brettanomyces* invertases constrained to: the closest eukaryote invertase clade (*b*), the more distant eukaryote invertase clade (*c*), and the eukaryote invertases within the alternate prokaryote clade (*d*). Tree topology tests are reported in Table S3.

## Data Availability

All sequencing reads and genome assemblies have been deposited at the National Center for Biotechnology Information (NCBI) Sequence Read Archive (SRA) under the BioProject: PRJNA554210. Raw FAST5-format files for all Oxford Nanopore sequencing are available from the European Bioinformatics Institute (EMBL-EBI) European Nucleotide Archive (ENA) under the study: ERP116386.

## Abbreviations

BGs: BUSCO Genes (the genes identified by the BUSCO pipeline)
BUSCO: The name of the pipeline for detecting Benchmarking universal single-copy orthologs
EMBL-EBI: European Molecular Biology Laboratory European Bioinformatics Institute
ENA: European Nucleotide Archive (ENA)
H1/H2: Haplome 1/Haplome 2 (*B. anomalus*)
HGT: Horizontal gene transfer
LOH: Loss of heterozygosity
NCBI: National Center for Biotechnology Information
SCO: Single copy ortholog
SRA: Sequence Read Archive

## References

[1] Michel RH, McGovern PE, Badler VR (1992). Chemical evidence for ancient beer. Nature.

[2] Fay JC, Benavides JA (2005). Evidence for domesticated and wild populations of Saccharomyces cerevisiae. PLoS Genet.

[3] Edgardo A, Carolina P, Manuel R, Juanita F, Baeza J (2008). Selection of thermotolerant yeast strains Saccharomyces cerevisiae for bioethanol production. Enzym Microb Technol.

[4] Marsit S, Dequin S. Diversity and adaptive evolution of Saccharomyces wine yeast: a review. FEMS Yeast Res. 2015;15(7):fov067. 10.1093/femsyr/fov067 PMC4629790 26205244

[5] Rozpędowska E, Hellborg L, Ishchuk OP, Orhan F, Galafassi S, Merico A, Woolfit M, Compagno C, Piškur J (2011). Parallel evolution of the make–accumulate–consume strategy in Saccharomyces and Dekkera yeasts. Nat Commun.

[6] Smith MT, Kurtzman CP, Fell JW, Boekhout T (2011). Chapter 89 - Brettanomyces Kufferath & van Laer (1921). The Yeasts (Fifth Edition).

[7] Peter G, Dlauchy D, Tobias A, Fulop L, Podgorsek M, Cadez N (2017). Brettanomyces acidodurans sp. nov., a new acetic acid producing yeast species from olive oil. Antonie Van Leeuwenhoek.

[8] Yamada Y, Matsuda M, Maeda K, Mikata K (1994). The phylogenetic relationships of species of the genus Dekkera van der Walt based on the partial sequences of 18S and 26S ribosomal RNAs (Saccharomycetaceae). Biosci Biotechnol Biochem.

[9] Yamada Y, Matsuda M, Mikata K (1995). The phylogenetic relationships ofEeniella nana Smith, Batenburg-van der Vegte et Scheffers based on the partial sequences of 18S and 26S ribosomal RNAs (Candidaceae). J Ind Microbiol.

[10] Röder C, König H, Fröhlich J (2007). Species-specific identification of Dekkera/Brettanomyces yeasts by fluorescently labeled DNA probes targeting the 26S rRNA. FEMS Yeast Res.

[11] Chatonnet P, Dubourdie D, Boidron J-N, Pons M (1992). The origin of ethylphenols in wines. J Sci Food Agric.

[12] Chatonnet P, Dubourdieu D, Boidron JN (1995). The Influence of *Brettanomyces/Dekkera* sp. Yeasts and Lactic Acid Bacteria on the Ethylphenol Content of Red Wines. Am J Enology Viticulture.

[13] Van Oevelen D, Spaepen M, Timmermans P, Verachtert H (1977). Microbiological aspects of spontaneous wort fermentation in the production of lambic and gueuze. J Inst Brew.

[14] Spaepen M, Van Oevelen D, Verachtert H (1978). Fatty acids and esters produced during the spontaneous fermentation of lambic and gueuze. J Inst Brew.

[15] Basso RF, Alcarde AR, Portugal CB (2016). Could non-Saccharomyces yeasts contribute on innovative brewing fermentations?. Food Res Int.

[16] De Souza Liberal AT, Basílio ACM, Do Monte Resende A, BTV B, Da Silva-Filho EA, JOF DM, Simões DA, De Morais MA (2007). Identification of Dekkera bruxellensis as a major contaminant yeast in continuous fuel ethanol fermentation. J Appl Microbiol.

[17] Reis ALS, de Fátima Rodrigues de Souza R, RRN BT, FCB L, PMG P, Vidal EE, de Morais MA (2014). Oxygen-limited cellobiose fermentation and the characterization of the cellobiase of an industrial Dekkera/Brettanomyces bruxellensis strain. SpringerPlus.

[18] Curtin CD, Borneman AR, Chambers PJ, Pretorius IS (2012). De-novo assembly and analysis of the heterozygous triploid genome of the wine spoilage yeast Dekkera bruxellensis AWRI1499. PLoS One.

[19] Albertin W, Panfili A, Miot-Sertier C, Goulielmakis A, Delcamp A, Salin F, Lonvaud-Funel A, Curtin C, Masneuf-Pomarede I (2014). Development of microsatellite markers for the rapid and reliable genotyping of Brettanomyces bruxellensis at strain level. Food Microbiol.

[20] Borneman AR, Zeppel R, Chambers PJ, Curtin CD (2014). Insights into the Dekkera bruxellensis genomic landscape: comparative genomics reveals variations in Ploidy and nutrient utilisation potential amongst wine isolates. PLoS Genet.

[21] Crauwels S, Zhu B, Steensels J, Busschaert P, De Samblanx G, Marchal K, Willems KA, Verstrepen KJ, Lievens B (2014). Assessing genetic diversity among genus-species Brettanomyces yeasts by DNA fingerprinting and whole-genome sequencing. Appl Environ Microbiol.

[22] Varela C, Lleixà J, Curtin C, Borneman A. Development of a genetic transformation toolkit for Brettanomyces bruxellensis. FEMS Yeast Res. 2018;18(7):foy070. 10.1093/femsyr/foy070 29982550

[23] Varela C, Bartel C, Roach M, Borneman A, Curtin C (2019). Brettanomyces bruxellensis SSU1 haplotypes confer different levels of sulfite tolerance when expressed in a Saccharomyces cerevisiae SSU1 null mutant. Appl Environ Microbiol.

[24] Fournier T, Gounot J-S, Freel K, Cruaud C, Lemainque A, Aury J-M, Wincker P, Schacherer J, Friedrich A (2017). High-Quality *de Novo* Genome Assembly of the *Dekkera bruxellensis* Yeast Using Nanopore MinION Sequencing. G3.

[25] Vervoort Y, Herrera-Malaver B, Mertens S, Guadalupe Medina V, Duitama J, Michiels L, Derdelinckx G, Voordeckers K, Verstrepen KJ (2016). Characterization of the recombinant Brettanomyces anomalus β-glucosidase and its potential for bioflavouring. J Appl Microbiol.

[26] Avramova M, Cibrario A, Peltier E, Coton M, Coton E, Schacherer J, Spano G, Capozzi V, Blaiotta G, Salin F (2018). Brettanomyces bruxellensis population survey reveals a diploid-triploid complex structured according to substrate of isolation and geographical distribution. Sci Rep.

[27] Hellborg L, Piskur J (2009). Complex nature of the genome in a wine spoilage yeast, Dekkera bruxellensis. Eukaryot Cell.

[28] Koren S, Phillippy AM (2015). One chromosome, one contig: complete microbial genomes from long-read sequencing and assembly. Curr Opin Microbiol.

[29] Jain M, Koren S, Miga KH, Quick J, Rand AC, Sasani TA, Tyson JR, Beggs AD, Dilthey AT, Fiddes IT (2018). Nanopore sequencing and assembly of a human genome with ultra-long reads. Nat Biotechnol.

[30] van der Walt J (1961). Brettanomyces custersianus Nov. spec. Antonie Van Leeuwenhoek.

[31] Kolfschoten GA, Yarrow D (1970). Brettanomyces naardenensis, a new yeast from soft drinks. Antonie Van Leeuwenhoek.

[32] Smith MT, van Grinsven AM (1984). Dekkera anomala sp. nov., the teleomorph of Brettanomyces anomalus, recovered from spoiled soft drinks. Antonie Van Leeuwenhoek.

[33] Boekhout T, Kurtzman CP, O'Donnell K, Smith MT (1994). Phylogeny of the yeast genera Hanseniaspora (anamorph Kloeckera), Dekkera (anamorph Brettanomyces), and Eeniella as inferred from partial 26S ribosomal DNA nucleotide sequences. Int J Syst Bacteriol.

[34] Cheng J, Guo X, Cai P, Cheng X, Piškur J, Ma Y, Jiang H, Gu Z (2017). Parallel evolution of chromatin structure underlying metabolic adaptation. Mol Biol Evol.

[35] Piskur J, Ling Z, Marcet-Houben M, Ishchuk OP, Aerts A, LaButti K, Copeland A, Lindquist E, Barry K, Compagno C (2012). The genome of wine yeast Dekkera bruxellensis provides a tool to explore its food-related properties. Int J Food Microbiol.

[36] Shen X-X, Opulente DA, Kominek J, Zhou X, Steenwyk JL, Buh KV, Haase MAB, Wisecaver JH, Wang M, Doering DT (2018). Tempo and Mode of Genome Evolution in the Budding Yeast Subphylum. Cell.

[37] Tiukova IA, Jiang H, Dainat J, Hoeppner MP, Lantz H, Piskur J, Sandgren M, Nielsen J, Gu Z, Passoth V (2019). Assembly and analysis of the genome sequence of the yeast Brettanomyces naardenensis CBS 7540. Microorganisms.

[38] Roach MJ, Schmidt SA, Borneman AR (2018). Purge Haplotigs: allelic contig reassignment for third-gen diploid genome assemblies. BMC Bioinformatics.

[39] Reuber BE, Karl C, Reimann SA, Mihalik SJ, Dodt G (1997). Cloning and functional expression of a mammalian gene for a Peroxisomal Sarcosine oxidase. J Biol Chem.

[40] Wagner MA, Jorns MS (2000). Monomeric Sarcosine oxidase: 2. Kinetic studies with Sarcosine, alternate substrates, and a substrate analogue. Biochemistry.

[41] Yoshida N, Akazawa S-I, Katsuragi T, Tani Y (2004). Characterization of two fructosyl-amino acid oxidase homologs of Schizosaccharomyces pombe. J Biosci Bioeng.

[42] Nishiya Y, Nakano S, Kawamura K (2012). Monomeric sarcosine oxidase acts on both L- and D-substrates. 生物試料分析.

[43] Qin Q-L, Xie B-B, Zhang X-Y, Chen X-L, Zhou B-C, Zhou J, Oren A, Zhang Y-Z (2014). A proposed genus boundary for the prokaryotes based on genomic insights. J Bacteriol.

[44] Dunn B, Richter C, Kvitek DJ, Pugh T, Sherlock G (2012). Analysis of the Saccharomyces cerevisiae pan-genome reveals a pool of copy number variants distributed in diverse yeast strains from differing industrial environments. Genome Res.

[45] Gallone B, Steensels J, Prahl T, Soriaga L, Saels V, Herrera-Malaver B, Merlevede A, Roncoroni M, Voordeckers K, Miraglia L (2016). Domestication and Divergence of *Saccharomyces cerevisiae* Beer Yeasts. Cell.

[46] Gonçalves M, Pontes A, Almeida P, Barbosa R, Serra M, Libkind D, Hutzler M, Gonçalves P, Sampaio José P (2016). Distinct domestication trajectories in top-fermenting beer yeasts and wine yeasts. Curr Biol.

[47] Harris V, Ford CM, Jiranek V, Grbin PR (2009). Survey of enzyme activity responsible for phenolic off-flavour production by Dekkera and Brettanomyces yeast. Appl Microbiol Biotechnol.

[48] Ault RG, Newton R, Findlay WPK (1971). Spoilage Organisms in Brewing. Modern Brewing Technology.

[49] Conterno L, Joseph CML, Arvik TJ, Henick-Kling T, Bisson LF (2006). Genetic and physiological characterization of *Brettanomyces bruxellensis* strains isolated from wines. Am J Enol Vitic.

[50] Woolfit M, Rozpędowska E, Piškur J, Wolfe KH (2007). Genome survey sequencing of the wine spoilage yeast *Dekkera (Brettanomyces) bruxellensis*. Eukaryot Cell.

[51] Parente DC, Cajueiro DBB, Moreno ICP, Leite FCB, De Barros PW, De Morais Jr MA (2018). On the catabolism of amino acids in the yeast Dekkera bruxellensis and the implications for industrial fermentation processes. Yeast.

[52] Ough CS, Stashak RM (1974). Further studies on Proline concentration in grapes and wines. Am J Enol Vitic.

[53] Jin H, Ferguson K, Bond M, Kavanagh T, Hawthorne D (1996). Malt nitrogen parameters and yeast fermentation behaviour. Proceedings of the convention-institute of brewing asia pacific section.

[54] Gorinstein S, Zemser M, Vargas-Albores F, Ochoa JL, Paredes-Lopez O, Scheler C, Salnikow J, Martin-Belloso O, Trakhtenberg S (1999). Proteins and amino acids in beers, their contents and relationships with other analytical data. Food Chem.

[55] Crauwels S, Van Assche A, de Jonge R, Borneman AR, Verreth C, Troels P, De Samblanx G, Marchal K, Van de Peer Y, Willems KA (2015). Comparative phenomics and targeted use of genomics reveals variation in carbon and nitrogen assimilation among different Brettanomyces bruxellensis strains. Appl Microbiol Biotechnol.

[56] Marsit S, Sanchez I, Galeote V, Dequin S (2016). Horizontally acquired oligopeptide transporters favour adaptation of Saccharomyces cerevisiae wine yeast to oenological environment. Environ Microbiol.

[57] Camarasa C, Bigey F, Marsit S, Dequin S, Nidelet T, Galeote V, Legras J-L, Sanchez I, Couloux A, Guy J (2018). Adaptation of S. cerevisiae to fermented food environments reveals remarkable genome plasticity and the footprints of domestication. Mol Biol Evol.

[58] Feurtey A, Stukenbrock EH (2018). Interspecific gene exchange as a driver of adaptive evolution in Fungi. Annu Rev Microbiol.

[59] Hall C, Brachat S, Dietrich FS (2005). Contribution of horizontal gene transfer to the evolution of *Saccharomyces cerevisiae*. Eukaryot Cell.

[60] Lin Z, Li W-H (2010). Expansion of hexose transporter genes was associated with the evolution of aerobic fermentation in yeasts. Mol Biol Evol.

[61] Monerawela C, James TC, Bond U, Wolfe KH. Loss of lager specific genes and subtelomeric regions define two different *Saccharomyces cerevisiae* lineages for Saccharomyces pastorianus Group I and II strains. FEMS Yeast Res. 2015;15(2):fou008. 10.1093/femsyr/fou008 25673756

[62] Steenwyk J, Rokas A (2017). Extensive Copy Number Variation in Fermentation-Related Genes Among *Saccharomyces cerevisiae* Wine Strains. G3.

[63] Yue J-X, Li J, Aigrain L, Hallin J, Persson K, Oliver K, Bergström A, Coupland P, Warringer J, Lagomarsino MC (2017). Contrasting evolutionary genome dynamics between domesticated and wild yeasts. Nat Genet.

[64] Chevreux B, Wetter T, Suhai S (1999). Genome sequence assembly using trace signals and additional sequence information. Computer Science and Biology: Proceedings of the German Conference on Bioinformatics (GCB) 99.

[65] Koren S, Schatz MC, Walenz BP, Martin J, Howard JT, Ganapathy G, Wang Z, Rasko DA, McCombie WR, Jarvis ED (2012). Hybrid error correction and de novo assembly of single-molecule sequencing reads. Nat Biotechnol.

[66] Koren S, Walenz BP, Berlin K, Miller JR, Bergman NH, Phillippy AM (2017). Canu: scalable and accurate long-read assembly via adaptive k-mer weighting and repeat separation. Genome Res.

[67] Li H (2018). Minimap2: pairwise alignment for nucleotide sequences. Bioinformatics (Oxford, England).

[68] Loman NJ, Quick J, Simpson JT (2015). A complete bacterial genome assembled de novo using only nanopore sequencing data. Nat Methods.

[69] Li H (2013). Aligning sequence reads, clone sequences and assembly contigs with BWA-MEM.

[70] Langmead B, Salzberg SL (2012). Fast gapped-read alignment with bowtie 2. Nat Methods.

[71] Walker BJ, Abeel T, Shea T, Priest M, Abouelliel A, Sakthikumar S, Cuomo CA, Zeng Q, Wortman J, Young SK (2014). Pilon: an integrated tool for comprehensive microbial variant detection and genome assembly improvement. PLoS One.

[72] Koboldt DC, Chen K, Wylie T, Larson DE, McLellan MD, Mardis ER, Weinstock GM, Wilson RK, Ding L. VarScan: variant detection in massively parallel sequencing of individual and pooled samples. Bioinformatics (Oxford, England). 2009;25(17):2283–5. 10.1093/bioinformatics/btp373 PMC2734323 19542151

[73] Edge P, Bafna V, Bansal V (2017). HapCUT2: robust and accurate haplotype assembly for diverse sequencing technologies. Genome Res.

[74] Patterson M, Marschall T, Pisanti N, van Iersel L, Stougie L, Klau GW, Schönhuth A (2015). WhatsHap: weighted haplotype assembly for future-generation sequencing reads. J Comput Biol.

[75] Kurtz S, Phillippy A, Delcher AL, Smoot M, Shumway M, Antonescu C, Salzberg SL (2004). Versatile and open software for comparing large genomes. Genome Biol.

[76] Gurevich A, Tesler G, Vyahhi N, Saveliev V (2013). QUAST: quality assessment tool for genome assemblies. Bioinformatics (Oxford, England).

[77] Kriventseva EV, Zdobnov EM, Simão FA, Ioannidis P, Waterhouse RM (2015). BUSCO: assessing genome assembly and annotation completeness with single-copy orthologs. Bioinformatics (Oxford, England).

[78] Stanke M, Keller O, Gunduz I, Hayes A, Waack S, Morgenstern B (2006). AUGUSTUS: ab initio prediction of alternative transcripts. Nucleic Acids Res.

[79] Kanehisa M, Sato Y, Morishima K (2016). BlastKOALA and GhostKOALA: KEGG tools for functional characterization of genome and Metagenome sequences. J Mol Biol.

[80] Jones P, Binns D, Chang H-Y, Fraser M, Li W, McAnulla C, McWilliam H, Maslen J, Mitchell A, Nuka G (2014). InterProScan 5: genome-scale protein function classification. Bioinformatics (Oxford, England).

[81] Emms DM, Kelly S (2015). OrthoFinder: solving fundamental biases in whole genome comparisons dramatically improves orthogroup inference accuracy. Genome Biol.

[82] Edgar RC (2004). MUSCLE: multiple sequence alignment with high accuracy and high throughput. Nucleic Acids Res.

[83] Suyama M, Torrents D, Bork P (2006). PAL2NAL: robust conversion of protein sequence alignments into the corresponding codon alignments. Nucleic Acids Res.

[84] Nguyen L-T, Schmidt HA, von Haeseler A, Minh BQ (2015). IQ-TREE: a fast and effective stochastic algorithm for estimating maximum-likelihood phylogenies. Mol Biol Evol.

[85] Krzywinski M, Schein J, Birol İ, Connors J, Gascoyne R, Horsman D, Jones SJ, Marra MA (2009). Circos: an information aesthetic for comparative genomics. Genome Res.

[86] Heymans K, Kuiper M, Maere S (2005). BiNGO: a Cytoscape plugin to assess overrepresentation of Gene Ontology categories in Biological Networks. Bioinformatics (Oxford, England).

[87] Gouy M, Gascuel O, Guindon S (2009). SeaView version 4: a multiplatform graphical user Interface for sequence alignment and phylogenetic tree building. Mol Biol Evol.

[88] Pruitt KD, Tatusova T, Klimke W, Maglott DR (2009). NCBI reference sequences: current status, policy and new initiatives. Nucleic Acids Res.

[89] Alexander WG, Wisecaver JH, Rokas A, Hittinger CT (2016). Horizontally acquired genes in early-diverging pathogenic fungi enable the use of host nucleosides and nucleotides. Proc Natl Acad Sci U S A.

