# Supplementary material for: New genome assemblies reveal patterns of domestication and adaptation across Brettanomyces (Dekkera) species
Source: BMC Genomics. 2020 Mar 2;21:194. doi: 10.1186/s12864-020-6595-z (PMC7052964; doi:10.1186/s12864-020-6595-z)
Supplement: Supplementary file 1 — Additional file 1: Table S1. MinION sequencing metrics for Brettanomyces sequencing, Table S2: Predicted genes, gene density, and orthogroup duplicity for the Brettanomyces genomes, Table S3: Constrained tree topology tests for Brettanomyces invertases (depicted in Figure S4), Table S4: Saccharomycetaceae species used with Brettanomyces species in OrthoFinder [file 12864_2020_6595_MOESM1_ESM.pdf]

Contents

Table S1: MinION sequencing metrics for *Brettanomyces* sequencing....1
Table S2: Predicted genes, gene density, and orthogroup duplicity for the *Brettanomyces* genomes....1
Table S3: Constrained tree topology tests for *Brettanomyces* invertases (depicted in Figure S4)....1
Table S4: Saccharomycetaceae species used with *Brettanomyces* species in OrthoFinder....2
References....3

Table S1: MinION sequencing metrics for *Brettanomyces* sequencing

	Median read length (bp)	Median read quality (QV)	Number of reads	Read length N50 (bp)	Total bases (Gb)
B. anomalus	4 685	8.7	296 479	7 859	1.65
B. custersianus	6 695	7.9	180 237	10 588	1.35
B. naardenensis	4 249	8.9	83 581	9 336	0.497
B. nanus	14 912	10.8	28 586	30 870	0.508

Table S2: Predicted genes, gene density, and orthogroup duplicity for the *Brettanomyces* genomes

	Number of genes	Total genic sequence (bp)	Total genic sequence (% of genome)	Orthogroups with multiple genes (% of orthogroups)
B. anomalus	5 735	8 571 724	62.2	10.4*
B. bruxellensis	5 293	8 469 775	64.2	6.0
B. custersianus	5 255	8 094 938	75.4	5.4
B. naardenensis	5 334	8 393 268	75.2	5.6
B. nanus	5 083	7 960 011	78.1	5.2
***S. cerevisiae* (S288C)**	6 445	9 008 924	74.1	9.7

* Averaged between H1 and H2

Table S3: Constrained tree topology tests for *Brettanomyces* invertases (depicted in Figure S4)

Tree	logL	deltaL	Bp-RELL	p-KH	p-SH	c-ELW	p-AU
**a**	-112583.8414	0	1	1	1	1	1
**b**	-112793.0893	209.25	0	0	0	6.92e-51	3.59e-06
**c**	-112833.7503	249.91	0	0	0	2.55e-69	4.67e-08
**d**	-112818.1804	234.34	0	0	0	1.86e-62	4.28e-34

Columns are as follows: Tree, the tree depicted in Figure S4; logL, the log likelihood; deltaL, the difference from the maximal logL in the set; bp-RELL, bootstrap proportion using RELL method (Kishino et al., 1990); p-KH, p-value of one sided Kishino-Hasegawa test (Kishino and Hasegawa, 1989); p-SH, p-value of Shimodaria-Hasegawa test (Ota et al., 2000); c-ELW, Expected likelihood weight (Strimmer and Rambaut, 2002); p-AU, p-value of approximately unbiased (AU) test (Shimodaira, 2002).

Table S4: Saccharomycetaceae species used with *Brettanomyces* species in OrthoFinder

**Species**	**Genome accession**
Agaricus bisporus	GCF_000300575.1
Babjeviella inositovora	GCF_001661335.1
Candida albicans	GCF_000182965.3
Candida boidinii	GCA_001599335.1
Candida glabrata	GCF_000002545.3
Candida tenuis	GCF_000002545.3
Citeromyces matritensis	GCA_003243085.1
Debaryomyces hansenii	GCF_000006445.2
Eremothecium gossypii	GCF_000091025.4
Hansenisapora osmophila	GCA_001747045.1
Kazachstania africana	GCF_000304475.1
Kluveromyces lactis	GCF_000002515.2
Komagataella phaffii	GCF_000027005.1
Lachancea thermotolerans	GCF_000142805.1
Lodderomyces elongisporus	GCF_000149685.1
Nadsonia fulvescens	GCA_001661315.1
Nakaseomyces bscillisporus	GCA_001046975.1
Naumovozyma dairenensis	GCF_000227115.2
Ogataea methanolica	GCA_001600755.1
Ogataea parapolymorpha	GCF_000187245.1
Ogataea polymorpha	GCF_001664045.1
Pachysolen tannophilus	GCA_001661245.1
Pichia membranifaciens	GCF_001661235.1
Saccharomyces bayanus	GCA_000167035.1
Saccharomyces boulardii	GCA_001413975.1
Saccharomyces cerevisiae	GCF_000146045.2
Saccharomyces eubayanus	GCF_001298625.1
Saccharomyces mikatae	GCA_000166975.1
Saccharomyces paradoxus	GCA_002079145.1
Saccharomyces uvarum	GCA_002242645.1
Spathaspora xylofermentans	GCA_002105455.1
Torulaspora delbrueckii	GCF_000243375.1
Wickerhamiella sorbophila	GCF_002251995.1
Wickerhamomyces anomalus	GCF_001661255.1
Wickerhamomyces ciferrii	GCF_000313485.1
Zygosaccharomyces parabailii	GCA_001984395.2

References

KISHINO, H. & HASEGAWA, M. 1989. Evaluation of the maximum likelihood estimate of the evolutionary tree topologies from DNA sequence data, and the branching order in hominoidea. *J Mol Evol,* 29**,** 170-9.

KISHINO, H., MIYATA, T. & HASEGAWA, M. 1990. Maximum likelihood inference of protein phylogeny and the origin of chloroplasts. *Journal of Molecular Evolution,* 31**,** 151-160.

OTA, R., WADDELL, P. J., HASEGAWA, M., SHIMODAIRA, H. & KISHINO, H. 2000. Appropriate Likelihood Ratio Tests and Marginal Distributions for Evolutionary Tree Models with Constraints on Parameters. *Molecular Biology and Evolution,* 17**,** 798-803.

SHIMODAIRA, H. 2002. An approximately unbiased test of phylogenetic tree selection. *Syst Biol,* 51**,** 492-508.

STRIMMER, K. & RAMBAUT, A. 2002. Inferring confidence sets of possibly misspecified gene trees. *Proceedings. Biological sciences,* 269**,** 137-142.
