# Supplementary figures and images for: New genome assemblies reveal patterns of domestication and adaptation across Brettanomyces (Dekkera) species
Source: BMC Genomics. 2020 Mar 2;21:194. doi: 10.1186/s12864-020-6595-z (PMC7052964; doi:10.1186/s12864-020-6595-z)

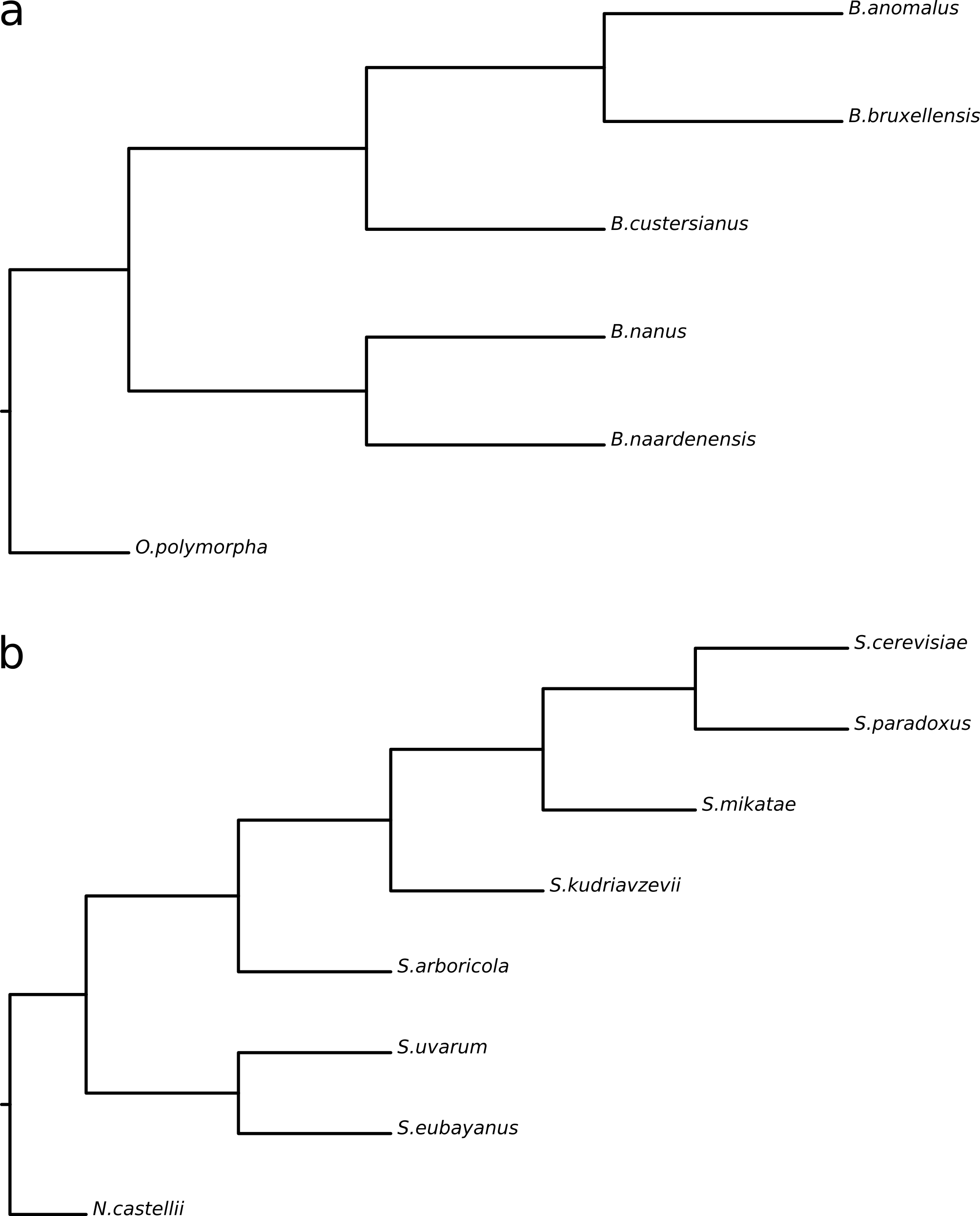

Supplement: Supplementary file 2 — Additional file 2: Figure S1. Coalescences-based phylogenies of Brettanomyces and Saccharomyces. Rooted, unscaled coalescence-based phylogenies were calculated from individual gene trees of all single copy orthologs for the Brettanomyces genus + Ogataea polymorpha (a), and for the Saccharomyces genus + Naumovozyma castellii (b). [file 12864_2020_6595_MOESM2_ESM.tiff]

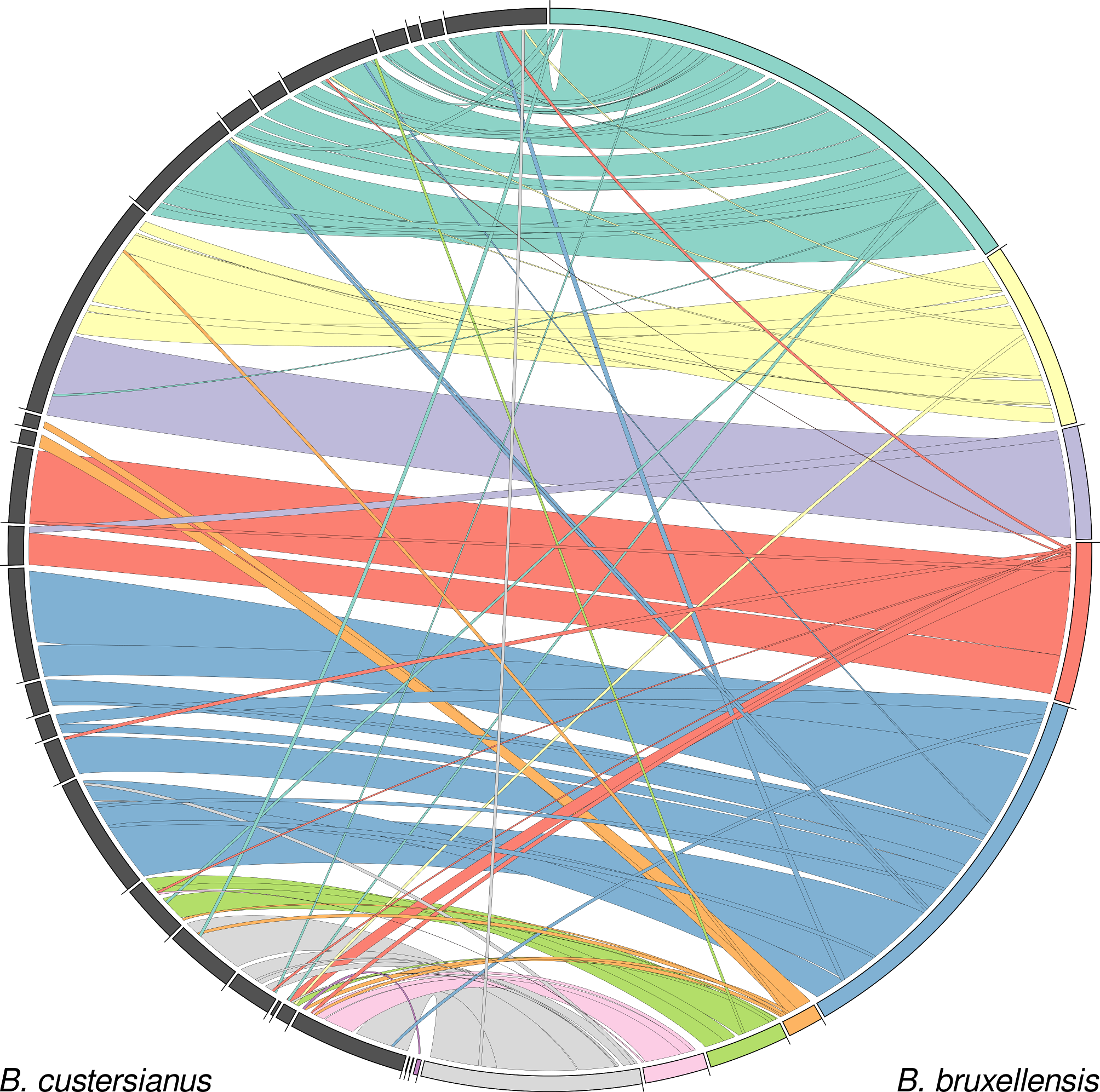

Supplement: Supplementary file 3 — Additional file 3: Figure S2. Synteny between haploid assemblies of B. bruxellensis and B. custersianus, visualized as a Circos plot. Reference assembly Contigs are coloured sequentially. Alignments are coloured according to the reference assembly contigs and are layered by alignment length. The query assembly contigs are coloured grey. [file 12864_2020_6595_MOESM3_ESM.tiff]

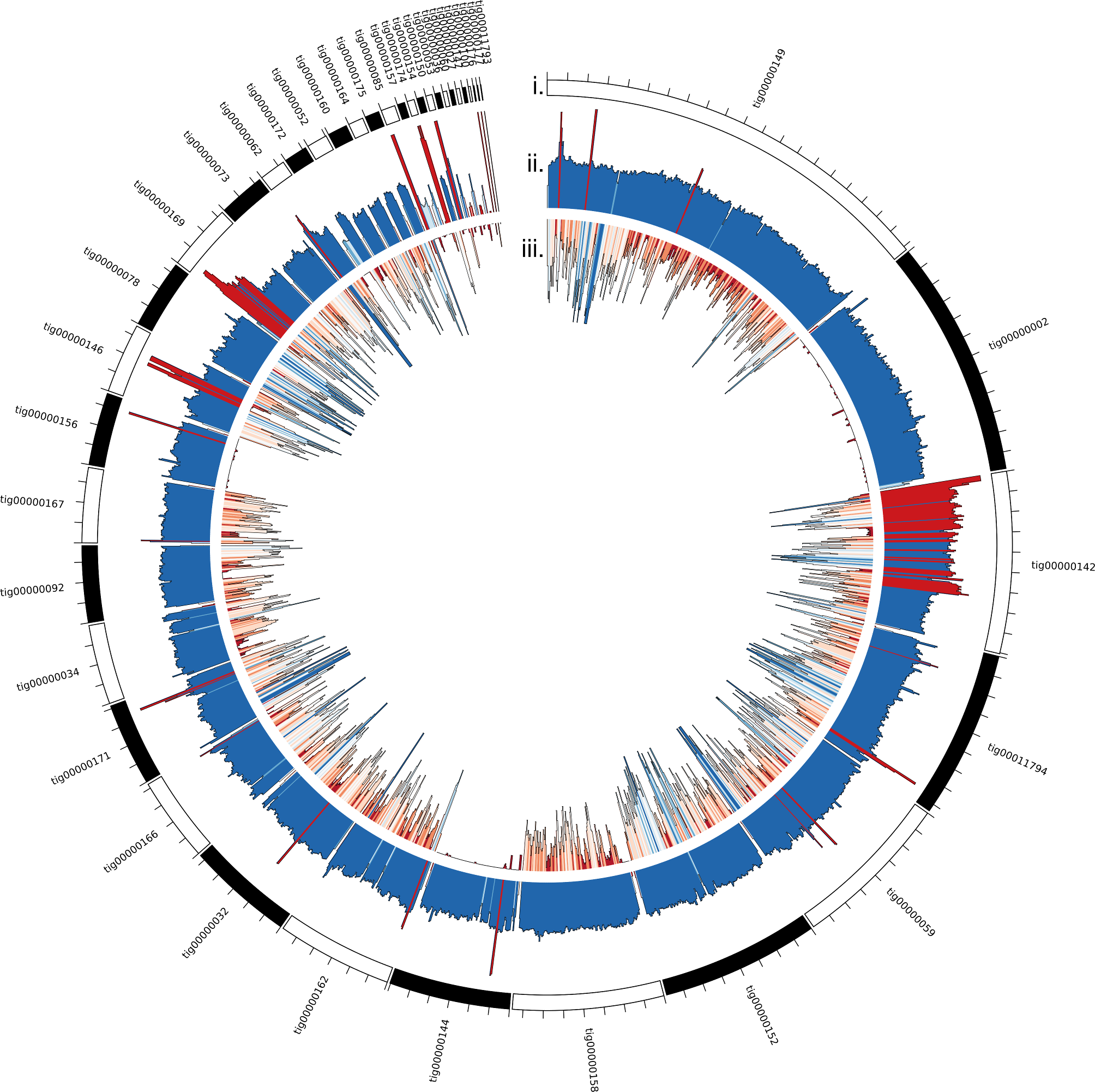

Supplement: Supplementary file 4 — Additional file 4: Figure S3. Read-depth and SNP density over haploid assembly of B. anomalus, visualized as a Circos plot. Contigs arranged by length (i), read-coverage histogram (blue, median coverage; red, low/high coverage) (ii), SNP-density (red, low; blue, high) (iii). [file 12864_2020_6595_MOESM4_ESM.tiff]

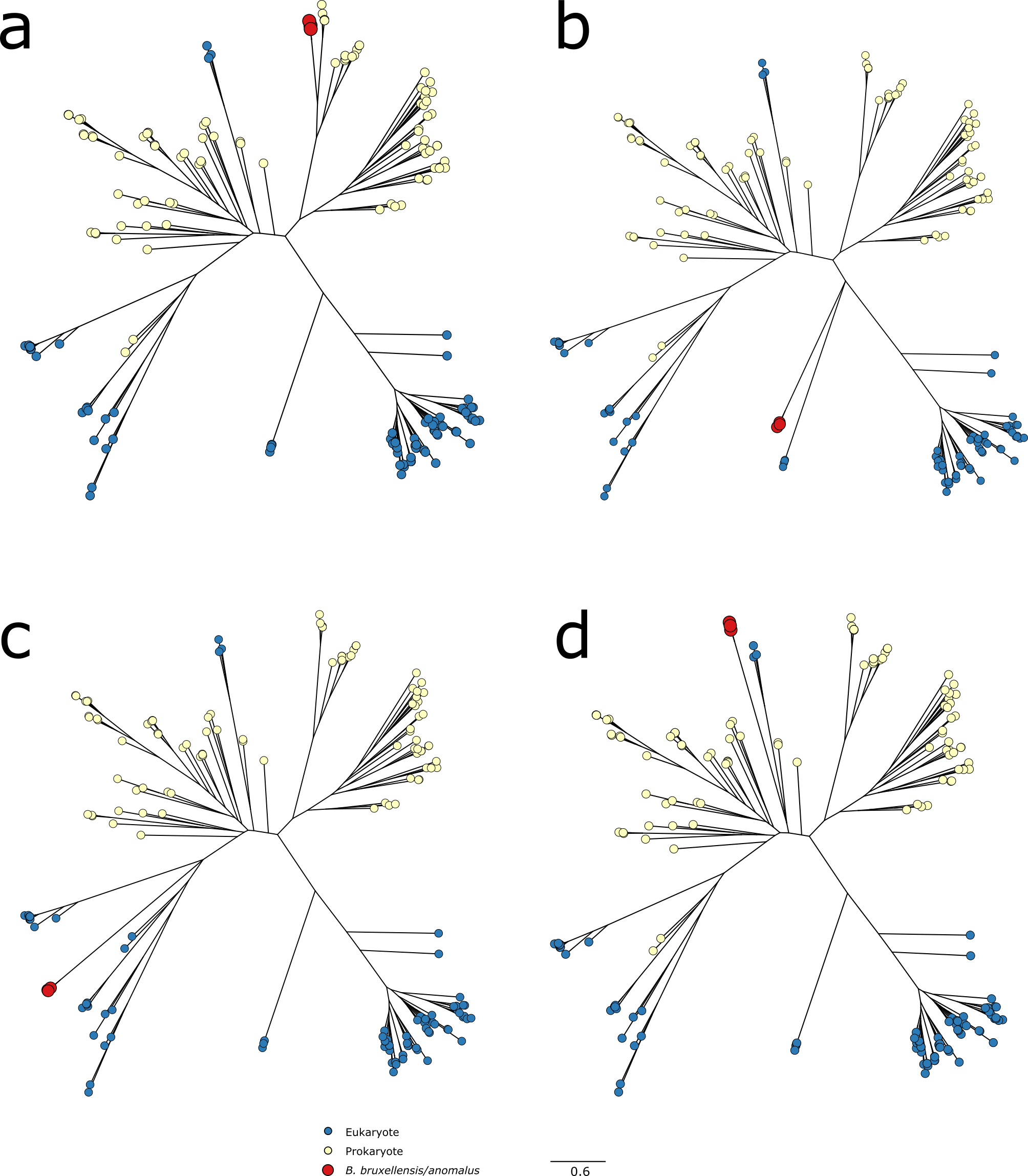

Supplement: Supplementary file 5 — Additional file 5: Figure S4. Constrained topology tests for Brettanomyces invertase genes. The unconstrained phylogeny (depicted in Fig. 4) of invertases from Brettanomyces and the top blast hits from the RefSeq non-redundant prokaryote and eukaryote databases, scaled by substitutions per site, with Brettanomyces nodes enlarged for clarity (a). Brettanomyces invertases constrained to: the closest eukaryote invertase clade (b), the more distant eukaryote invertase clade (c), and the eukaryote invertases within the alternate prokaryote clade (d). Tree topology tests are reported in Table S3. [file 12864_2020_6595_MOESM5_ESM.tiff]
